# SOX2 regulates homeostasis of taste bud cells and lingual epithelial cells in posterior tongue

**DOI:** 10.1371/journal.pone.0240848

**Published:** 2020-10-15

**Authors:** Makoto Ohmoto, Weiwei Lei, Junpei Yamashita, Junji Hirota, Peihua Jiang, Ichiro Matsumoto

**Affiliations:** 1 Monell Chemical Senses Center, Philadelphia, PA, United States of America; 2 Center for Biological Resources and Informatics, Tokyo Institute of Technology, Yokohama, Kanagawa, Japan; 3 Department of Life Science and Technology, Tokyo Institute of Technology, Yokohama, Kanagawa, Japan; Duke University, UNITED STATES

## Abstract

Taste bud cells arise from local epithelial stem cells in the oral cavity and are continuously replaced by newborn cells throughout an animal’s life. However, little is known about the molecular and cellular mechanisms of taste cell turnover. Recently, it has been demonstrated that SOX2, a transcription factor expressed in epithelial stem/progenitor cells of the oral cavity, regulates turnover of anterior tongue epithelium including gustatory and non-gustatory papillae. Yet, the role of SOX2 in regulating taste cell turnover in the posterior tongue is unclear. Prompted by the fact that there are regional differences in the cellular and molecular composition of taste buds and stem/progenitor cells in the anterior and posterior portions of tongue, which are derived from distinct embryonic origins, we set out to determine the role of SOX2 in epithelial tissue homeostasis in the posterior tongue. Here we report the differential requirement of SOX2 in the stem/progenitor cells for the normal turnover of lingual epithelial cells in the posterior tongue. *Sox2* deletion in the stem/progenitor cells neither induced active caspase 3-mediated apoptotic cell death nor altered stem/progenitor cell population in the posterior tongue. Nevertheless, morphology and molecular feature of non-gustatory epithelial cells were impaired in the circumvallate papilla but not in the filiform papillae. Remarkably, taste buds became thinner, collapsed, and undetectable over time. Lineage tracing of Sox2-deleted stem/progenitor cells demonstrated an almost complete lack of newly generated basal precursor cells in the taste buds, suggesting mechanistically that *Sox2* is involved in determining stem/progenitor cells to differentiate to gustatory lineage cells. Together, these results demonstrate that SOX2 plays key roles in regulating epithelial tissue homeostasis in the posterior tongue, similar but not identical to its function in the anterior tongue.

## Introduction

Taste buds comprise tens of cells, including taste receptor cells, to sense different taste qualities [1–3]. In the dorsal tongue of mice, they are localized in the papillary structures, fungiform, foliate, and circumvallate papillae. Fungiform papillae (FuP) are scatterd in the anterior two-thirds of dorsal tongue and house single taste buds, whereas circumvallate papilla (CvP) is located in the middle-line, proximal to the posterior end of tongue and house many taste buds in trench. Foliate papillae (FoP) are localized lateral side of dorsal tongue and house many taste buds as CvP. As such uneven distribution of papillae and taste buds, regional differences are observed for the population and distribution of taste cells. Umami taste cells that express umami taste receptor gene *Tas1r1* (originally named TR1) exist predominantly in the FuP in the anterior tongue and are rarely found in the CvP or FoP in the posterior tongue, whereas sweet taste cells expressing sweet taste receptor gene *Tas1r2* (originally named TR2) are rare in the FuP but many in the CvP and FoP [4, 5]. Sodium taste cells that express amiloride–sensitive sodium detecting mechanism exist in the FuP and probably palate but not in the posterior tongue [2].

Taste bud cells are short-lived, with average half lives of about a week, and are thus replenished continuously from local epithelial stem cells [6–8]. Stem cells in CvP are developmentally derived from endoderm and express *Lgr5*, while those in and/or around FuP are derived from ectoderm, express *Lgr5* in the neonatal mice, and cease the *Lgr5* expression in adult [9–11]. Like the distribution and frequency of taste buds and gene expression in taste bud cells, stem cells in the different regions of tongue epithelium may exhibit different properties.

SOX2 is one of the most extensively studied transcription factors, especially in research focused on embryonic and induced pluripotent stem cells and development such as organogenesis [12–14]. In adult mice, it is predominantly expressed in stem cells of epithelial tissues, including the oral cavity, and brain [15–21]. However, the contribution of SOX2 in stem cells to tissue homeostasis in adult mice differs among tissues. SOX2 is required for the normal turnover of neurons in the hippocampus and epithelial cells in trachea but is dispensable for the homeostasis of gastric and pulmonary epithelia [14, 16, 17]. In oral epithelia, especially in the anterior tongue, innate hypomorphic *Sox2* mutant mice have largely defective taste bud development, and SOX2 overexpression in putative non-gustatory stem/progenitor cells yields many FuP-like structures that seem to be devoid of taste buds [22]. *Sox2* deficiency in the tongue epithelial stem/progenitor cells increased the number of proliferating cells and blocked the differentiation of gustatory and non-gustatory epithelial cells in the FuP [23]. However, it remains unclear whether SOX2 plays the same role for the homeostasis of epithelial cells in the posterior tongue as in the anterior tongue.

In this study, we explored the roles of SOX2 in epithelial stem/progenitor cells in the posterior tongue. Upon deletion of *Sox2*, oral epithelial stem/progenitor cells in adult mice ceased to differentiate into taste bud cells in the CvP, manifested by a gradual decrease in the number of taste bud cells and an eventually complete loss of taste buds. Apoptosis was not induced, and the number and distribution of stem/progenitor cells in the CvP seemed to be unaltered. The expression of *Sprr2a2*, a marker of non-gustatory stratified squamous epithelial cells in the CvP were quickly decreased but not disappeared, suggesting that *Sox2*-deficient stem/progenitor cells continued to generate non-gustatory epithelial cells. No obvious changes were observed in gene expression patterns and morphology in filiform papillae (FiP), non-gustatory papillae in the tongue. Thus, SOX2 is required in taste stem/progenitor cells for maintaining homeostasis of taste buds, but is dispensable in the epithelial stem/progenitor cells outside CvP for maintaining homeostasis of non-gustatory papillary epithelium.

## Results

### Stem cell-specific *Sox2* deficiency leads to the loss of taste buds

**Fig 1:**
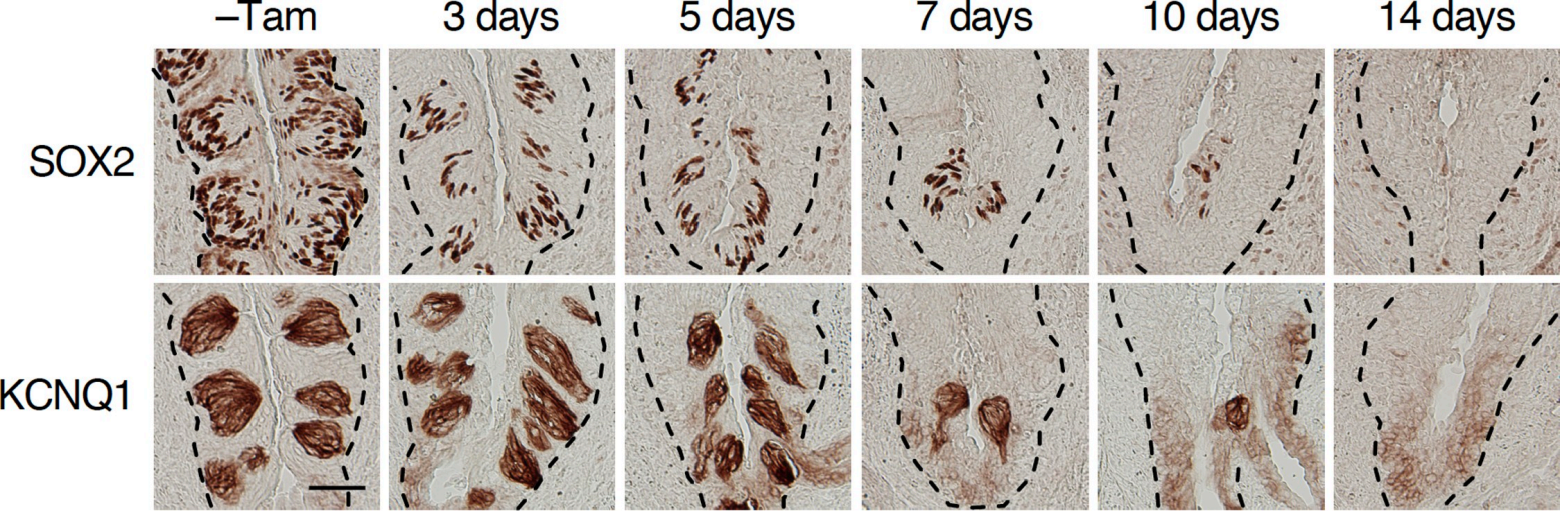
*Sox2* deletion in epithelial stem/progenitor cells results in the disappearance of taste buds. Immunohistochemistry to marker proteins of taste bud cells in the circumvallate papillae (CvP) of *Krt5*^*CreERT2/+*^; *Sox2*^*flox/flox*^ mice with and without tamoxifen injection (–Tam, control): SOX2 (*top*) and KCNQ1 (*bottom*) as a pan-taste bud cell marker. The broken lines show the boundary of epithelium and connective tissue. Number of analyzed mice was 3 at each point. Scale bar, 50 μm.

**Fig 2:**
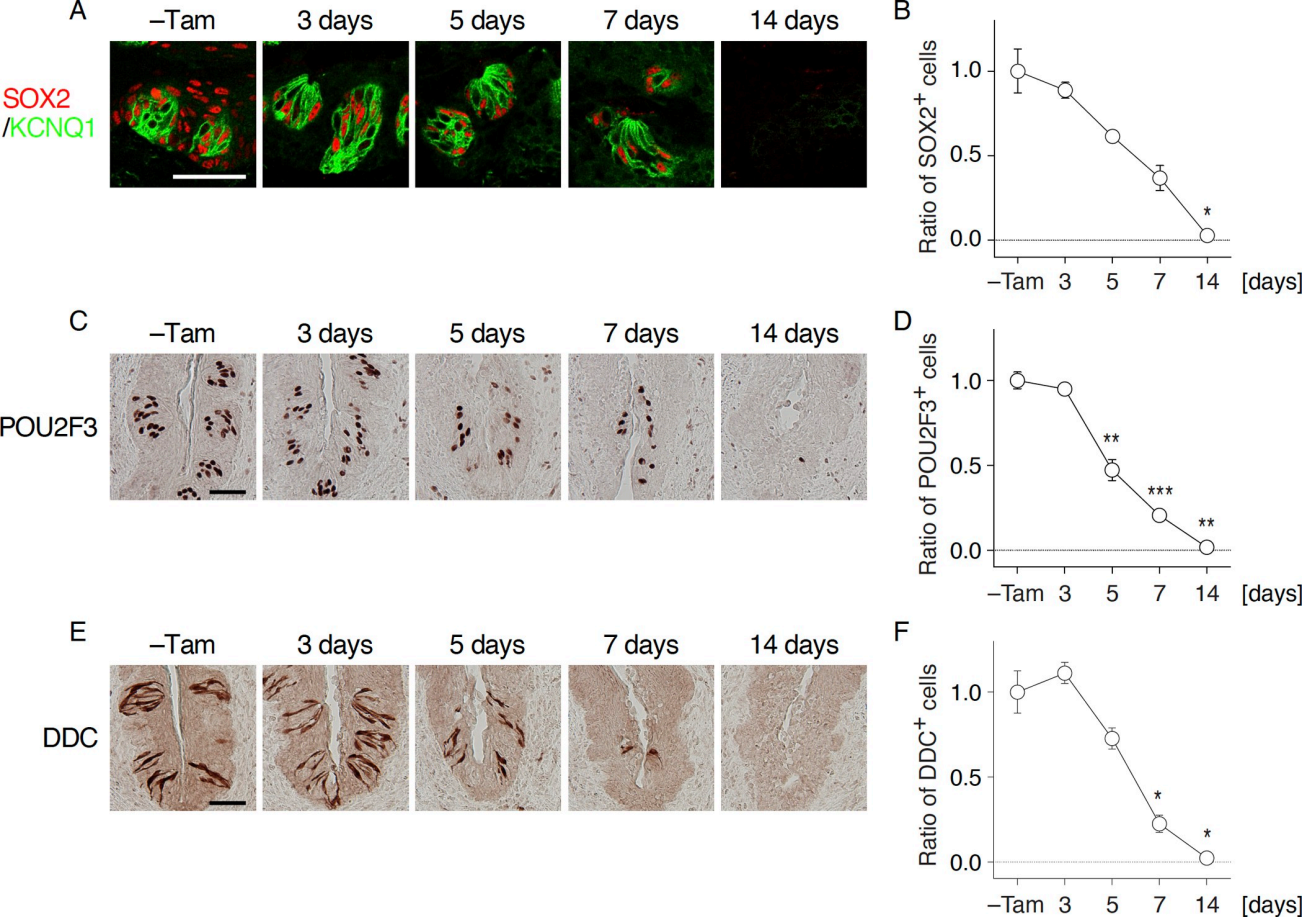
Taste bud cell populations are decreased after *Sox2* deletion in epithelial stem/progenitor cells. **A, C, E:** Immunohistochemical detection of taste bud cell marker SOX2 (non-sensory taste bud cells, A), POU2F3 (sweet, umami, and bitter taste cells, C), and DDC (sour taste cells, E) in the CvP of *Krt5*^*CreERT2/+*^; *Sox2*^*flox/flox*^ mice with and without tamoxifen injection (–Tam, control). Double fluorescent labeling of SOX2 (red) and KCNQ1 (green) was done to identify SOX2 immunoreactive signals inside taste buds. **B, D, F:** Quantitative analyses of SOX2^+^ (B), POU2F3^+^ (D), and DDC^+^ (F) cells in taste buds in the CvP. Immunoreactive signals were counted, and the ratios of signals after the induction of *Sox2* deletion to those in control (–Tam) per trench were statistically analyzed using Welch’s ANOVA to evaluate significant change over time (n = 3 at each time point). The data are expressed as the mean ± s.e.m. *p < 0.05, **p < 0.01, ***p < 0.001. Scale bars, 50 μm.

In the oral epithelium, SOX2 is expressed both in stem/progenitor cells and in a subset of taste bud cells that are continuously replenished from local *Sox2*^+^ epithelial stem/progenitor cells [18, 21, 22, 24]. To determine the role of SOX2 in the stem/progenitor cell population, a suitable Cre-driver strain to delete *Sox2* specifically in the stem/progenitor cells was required. *Krt5* is known to be a marker for stem/progentior cells in tongue epithelium. Indeed, *in situ* hybridization studies revealed that *Krt5* was predominantly expressed in the basal cells of oral epithelia, especially in PCNA^+^ cells that were also SOX2 immunoreactive, whereas it was not expressed in taste buds (S1A–S1C Fig). Lineage tracing studies using *Krt5*^*CreERT2/+*^; *Rosa26*^*loxP-stop-loxP-tdTomato/+*^ (*Rosa26*^*lsl-Tom/+*^) mice demonstrated that oral epithelia began to be populated with tdTomato^+^ cells shortly after the induction of tdTomato by tamoxifen injection and that tdTomato^+^ cells occupied the whole epithelia, including taste bud cells (S1D and S1E Fig). Although tamoxifen-untreated using *Krt5*^*CreERT2/+*^; *Rosa26*^*lsl-Tom/+*^ mice occasionally showed sporadic spontaneous tdTomato expression in tongue epithelium (S1F Fig), it is unrelated to *Sox2* deletion in stem/progenitor cells. Given the efficacy, we found *Krt5*^*CreERT2/+*^ strain useful to induce *Sox2* deletion specifically in stem/progenitor cells,

Shortly after tamoxifen injection, SOX2 immunoreactivity completely disappeared in non-gustatory oral epithelia surrounding CvP and robustly decreased in the epithelia of CvP in *Krt5*^*CreERT2/+*^; *Sox2*^*flox/flox*^ mice (Figs 1 and S2). The remaining SOX2 immunoreactivity in CvP was confined to taste bud cells, gradually decreased over time, and finally disappeared by 2 weeks after tamoxifen injection (Figs 1, 2A and 2B), consistent with the turnover rate of taste bud cells [6, 8]. Significant decrease of SOX2 signals in taste buds was observed 5 days after tamoxifen injection (5 days, *p* = 0.0108; 7 days, *p* = 0.0003; and 14 days, *p*<0.0001). In parallel with the over time decrease of SOX2 immunoreactive signals, KCNQ1 (a pan-taste cell marker [25]) immunoreactive signals that can be observed in almost all taste bud cells were also decreased and completely disappeared 14 days after tamoxifen injection (Fig 1), suggesting that taste bud cells were not regenerated after inducing *Sox2* deficiency in the stem/progenitor cells. Similarly, immunoreactive signals of POU2F3, a transcription factor specifying the sweet, umami, and bitter taste cell lineage [26], and DDC, a marker of sour taste cells [27], also decreased and then completely disappeared after *Sox2* ablation (POU2F3 at 5 days, 7 days, and 14 days, *p*<0.0001; DDC at 7 days and 14 days, *p*<0.0001) (Fig 2C–2F). These results suggest that *Sox2* is required in stem/progenitor cells to maintain taste buds in the gustatory papillae. It is worthy of noting that the significant decrease of immunoreactive signals to DDC is observed later than POU2F3, which is reminiscent of longer longevity of sour taste cells than sweet, umami, and bitter taste cells [8].

### Caspase 3-mediated apoptotic cell death is not induced during the decrease of taste bud cells by stem cell-specific *Sox2* deficiency

**Fig 3:**
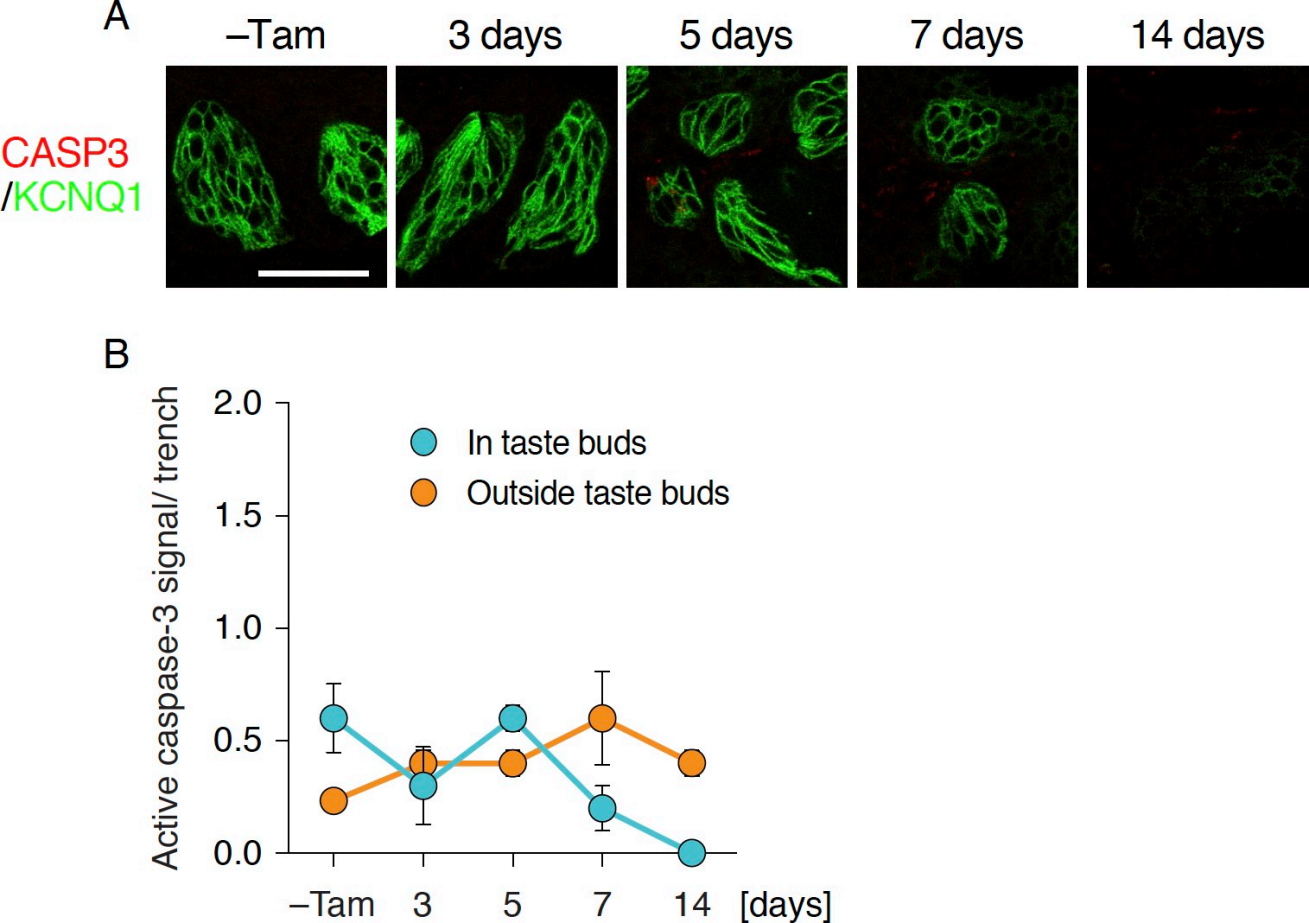
Unaltered apoptotic cell death after *Sox2* deletion in epithelial stem/progenitor cells. **A:** Double fluorescence immunohistochemical labeling of active CASP3 (red) and KCNQ1 (green) in CvP of *Krt5*^*CreERT2/+*^; *Sox2*^*flox/flox*^ mice with and without tamoxifen injection (–Tam, control). Scale bar, 50 μm. **B:** Quantitative analyses of active CASP3^+^ cells in (blue) and outside taste buds (orange). Numbers of active CASP3^+^ cells per trench wall were statistically analyzed using Welch’s ANOVA to evaluate significant change over time (n = 3 at each time point). The data are expressed as the mean ± s.e.m.

The decrease of taste bud cells by deleting *Sox2* in the stem/progenitor cell population could be explained by the accelerated death of *Sox2*-deleted taste bud cells despite continuous generation of new taste bud cells by stem/progenitor cells. Because acute non-cell autonomous cell death was observed in FuP by *Sox2* deficiency [23], non-cell autonomonous cell death may occur in the CvP. It is also possible that *Sox2*-deleted stem/progenitor cells ceased to differentiate into taste bud cells. To determine which may be the principal reason for taste bud degeneration after Sox2 ablation, we first examined if apoptotic cell death was induced in the CvP by immunohistochemical analyses using anti-active caspase-3 (CASP3) antibody. Immunoreactivity to active CASP3 was rarely observed throughout oral epithelia with and without the induction of stem/progenitor cell-specific *Sox2* deletion, and no significant increase of active CASP3 signals was observed at any time points (Fig 3). Considering that the number of taste bud cells were decreased gradually but not acutely, it is very likely that Casp3-mediated apoptotic cell death or non-cell autonomouse cell death was not induced by *Sox2* deficiency in epithelial stem/progenitor cells in CvP.

### *Sox2*-deficient epithelial stem/progenitor cells are incapable of producing taste bud cells

**Fig 4:**
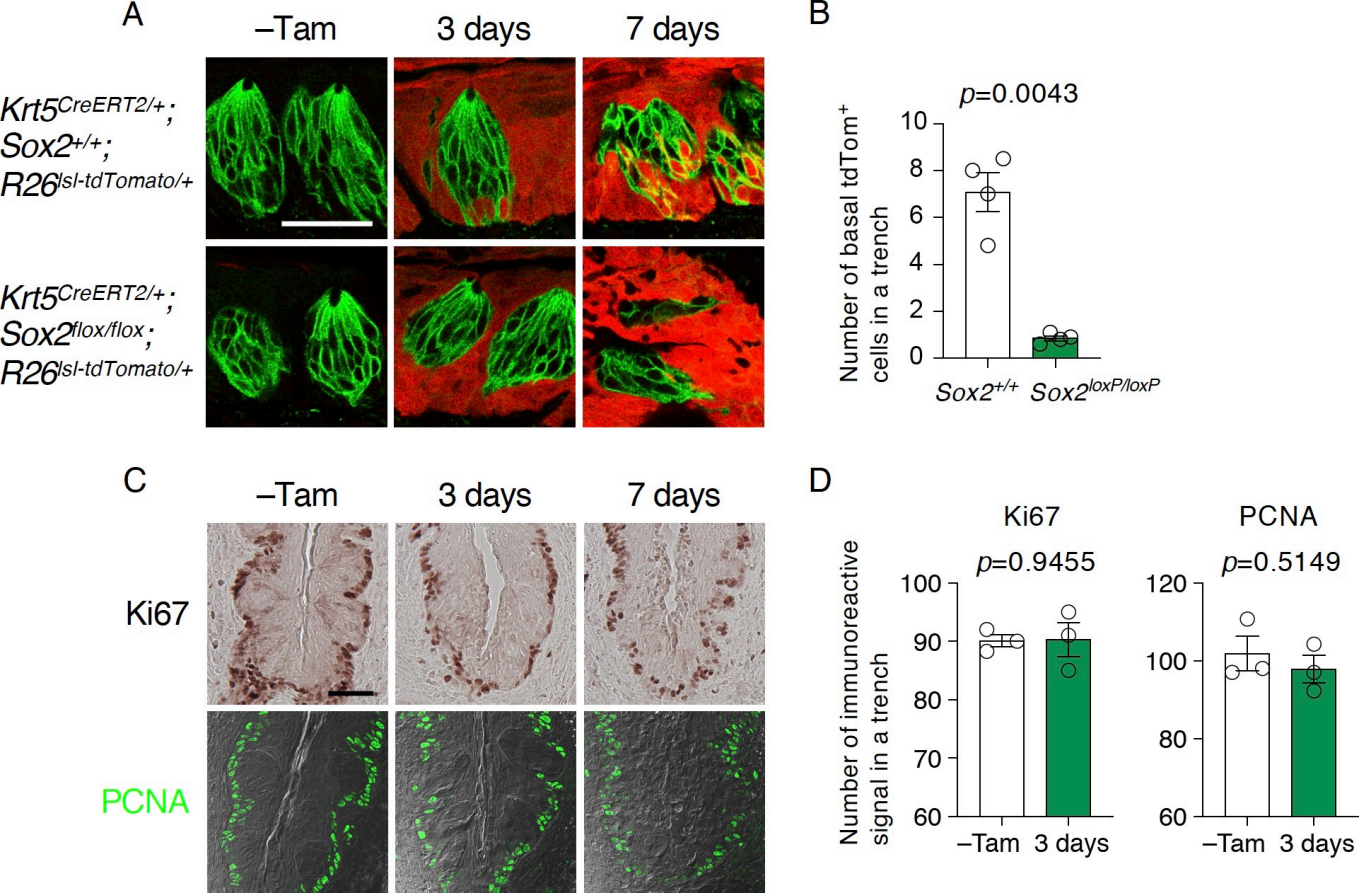
*Sox2*-deleted stem/progenitor cells are incapable of supplying new cells in taste buds. **A:** Lineage tracing of epithelial stem/progenitor cells in CvP of wild type (*top*) and *Sox2*-deleted mice (*bottom*). *Sox2* deletion was induced concominantly with the induction of tdTomato expression by tamoxifen into *Krt5*^*CreERT2/+*^; *Sox2*^*flox/flox*^; *Rosa26*^*lsl-Tom/+*^ mice. Taste buds were identified by KCNQ1 immunoreactivity (green). *Sox2* wild type mice,–Tam (n = 1), 3 days (n = 4), and 7 days (n = 2); *Sox2*-deleted mice,–Tam (control: no tamoxifen injection, n = 2), 3 days (n = 4), and 7 days (n = 2). **B:** Quantitative analyses of newly generated basal taste bud cells from wild type (*Krt5*^*CreERT2/+*^; *Sox2*^*+/+*^; *Rosa26*^*lsl-Tom/+*^) and *Sox2*-deleted (*Krt5*^*CreERT2/+*^; *Sox2*^*LoxP/LoxP*^ (cKO); *Rosa26*^*lsl-Tom/+*^) epithelial stem/progenitor cells. tdTomato^+^ basal cells in taste buds per trench were analyzed using Welch’s t-test to evaluate if the difference is statistically significant (n = 4). **C:** Immunohistochemical detection of Ki67 (*top*) and PCNA (*bottom*) in CvP of *Krt5*^*CreERT2/+*^; *Sox2*^*flox/flox*^ mice with and without tamoxifen injection (–Tam, control). **D:** Quantitative analyses of immunoreactive signals to Ki67 (*left*) and PCNA (*right*) in CvP trench of *Krt5*^*CreERT2/+*^; *Sox2*^*flox/flox*^ mice 3 days after tamoxifen injection and without tamoxifen injection (–Tam, control). Numbers of immunoreactive singals to proliferative cell markers per trench were statistically analyzed using Welch’s t-test (n = 3). Scale bars, 50 μm.

Gradual decrease of taste bud cells without acceleration or induction of cell death strongly suggests that the *Sox2* deletion affects taste bud cell generation. To determine if *Sox2* deletion impairs generation of taste bud cells, we conducted lineage tracing of *Sox2*-deleted epithelial stem/progenitor cells by concomitantly inducing *Sox2* deletion and tdTomato expression in them in *Krt5*^*CreERT2/+*^; *Rosa26*^*lsl-Tom/+*^*; Sox2*^*flox/flox*^ mice. In taste buds of the control mice having *Sox2* wild type alleles, tdTomato expression was first observed in the basal cells shortly after tamoxifen injection and then in the differentiated cells in taste buds, and the population of tdTomato^+^ taste bud cells increased over time after tamoxifen injection (Fig 4A). In contrast, we observed few tdTomato^+^ basal cells in taste buds of the stem cell-specific *Sox2*-deleted mice, while tdTomato expression was observed in the non-gustatory stratified squamous epithelial cells throughout CvP epithelium (Fig 4A and 4B). These results strongly suggest that *Sox2* deletion eliminates competency of stem/progenitor cells to differentiate into taste bud cells.

Futhermore, immunohistochemical analyses using antibodies against proliferating cell markers Ki67 and PCNA revealed no drastic or significant change in the distribution or frequency of proliferating (i.e. active stem) cells at the base of epithelium 3 days after tamoxifen injection, although proliferating cells appeared to be slightly disorganized 7 days after tamoxifen injection (Fig 4C and 4D). These results indicate that *Sox2* is not required for the viability of epithelial stem/progenitor cells. Partial disarrangement of stem/progenitor cell distribution can be explained by the structural changes resulting from a partial or complete loss of taste buds. Together, *Sox2* deficiency predominantly impairs the competency of stem/progenitor cells in the CvP to differentiate into gustatory cell lineage and is permissive for their competency to self-renew. A gradual decrease in the number of taste bud cells is likely due to the inability of stem/progenitor cells to replenish taste buds.

### *Sox2* deficiency impairs *Lgr5* expression in epithelial stem/progenitor cells

**Fig 5:**
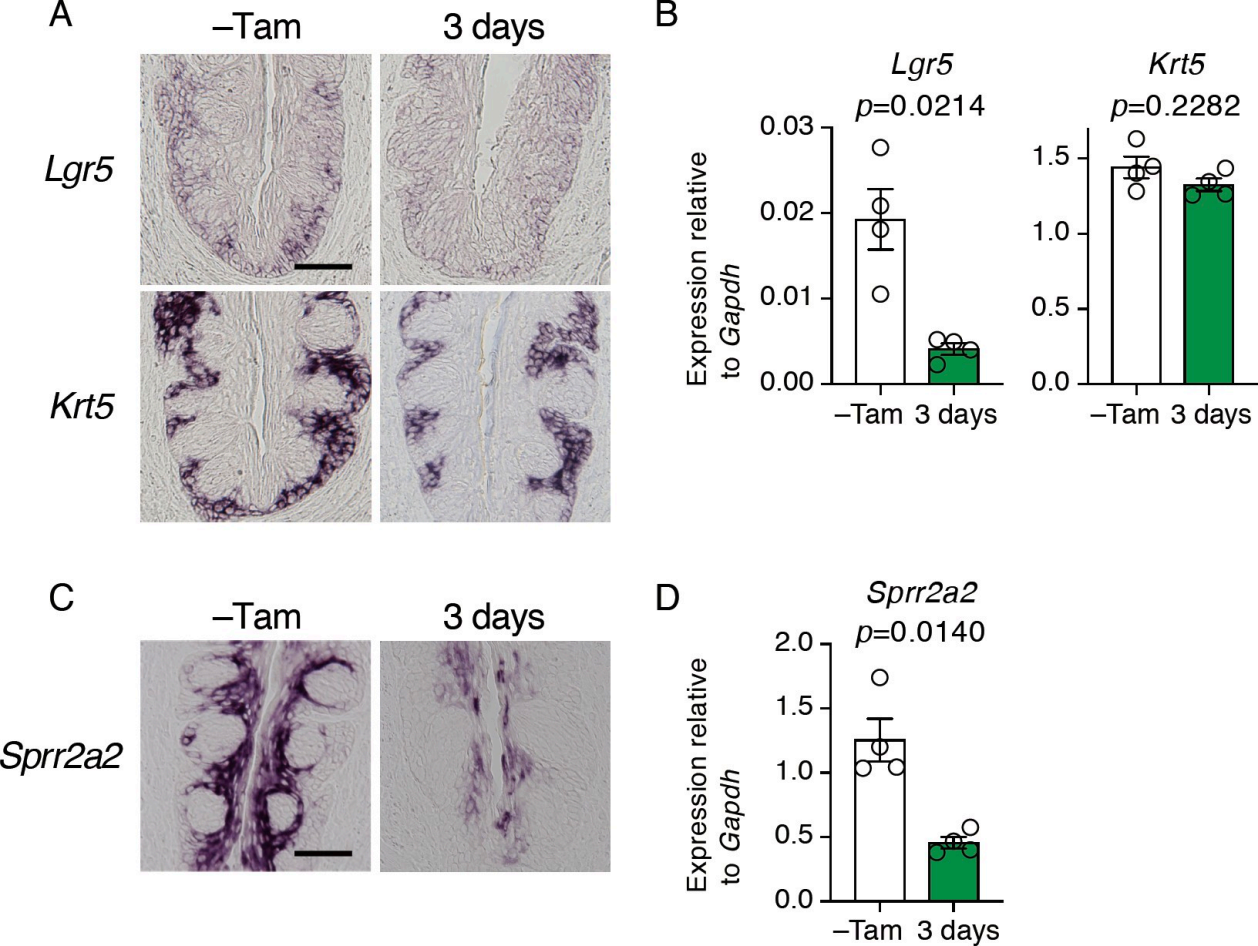
Impact of *Sox2* deletion in epithelial stem/progenitor cells on gene expression in stem/progenitor cells and non-gustatory epithelial cells. **A, C:** Qualitative histochemical analyses by *in situ* hybridization of stem/progenitor cell (A) and non-gustatory epithelial cell (C) genes in the CvP of *Krt5*^*CreERT2/+*^; *Sox2*^*flox/flox*^ mice (n≥3 at each point) before and after tamoxifen injection. **B, D:** Quantitative analyses of mRNA expression by PCR of *Lgr5* (B, *left*), *Krt5* (B, *right*), and *Sprr2a2* (D) in the CvP of *Krt5*^*CreERT2/+*^; *Sox2*^*flox/flox*^ mice before and after tamoxifen injection. Relative gene expression levels were normalized using *Gapdh* and statistically evaluated by Welch’s t-test (n = 4). Scale bars, 50 μm.

Development and differentiation of taste buds involve Wnt signaling [28–30]. In many eipthelial tissues, Lgr5 and its ligand R-Spondins can enhance the activity of Wnt signaling [31–33]. Because *Lgr5* is expressed in epithelial stem/progenitor cells in the CvP [10, 11], and *Sox2* deficiency prevented stem/progenitor cells from giving rise to taste bud cells, we asked if *Sox2* deletion impacts on *Lgr5* expression. In the CvP of the control mice, signals of *Lgr5* mRNA by *in situ* hybridization analyses were observed in the basal region of epithelium where *Krt5* expression was observed (Fig 5A). In the stem cell-specific *Sox2*-deficient mice, however, *Lgr5* signals were barely detectable, and quantitative RT-PCR revealed a significant decrease of *Lgr5* expression by *Sox2* deficiency, whereas *Krt5* expression was unaltered (Fig 5A and 5B). These results indicate that SOX2 directly or indirectly regulates *Lgr5* expression in the stem/progenitor cells, which may be involved in regulating taste bud cell generation.

### Anomalous non-gustatory epithelial cells are generated from *Sox2*-deficient stem/progenitor cells

Because non-gustatory epithelial cells surrounding taste buds in the CvP were continuously generated from *Sox2*-deleted stem/progenitor cells (Fig 4A and 4B), *Sox2* appears to be dispensable for the homeostasis of non-gustatory epithelial cells. However, it is unclear if *Sox2* deficiency will alter the expression profile or potentially the function of non-gustatory epithelial cells. To determine this, we then examined the impact of *Sox2* deficiency on the expression of *Sprr2a2* that is observed in the non-gustatory epithelial cells in the middle to apical area of trench of CvP where *Krt5* is not expressed (Fig 5C) [34]. Interestingly, *Sprr2a2* expression was significantly decreased and biased in the apical most area in the CvP shortly after *Sox2* deletion (Fig 5C and 5D). These results suggest that *Sprr2a2*^+^ cells in the apical area are derived from *Sox2*-expressing stem/progenitor cells, and that *Sox2*-deleted stem/progenitor cells continuously give rise to anomalous non-gustatory epithelial cells, albeit with the diminished expression of *Sprr2a2* in the CvP.

### Long-term impacts of *Sox2* deficiency in stem/progenitor cells on the homeostasis of CvP epithelium

**Fig 6:**
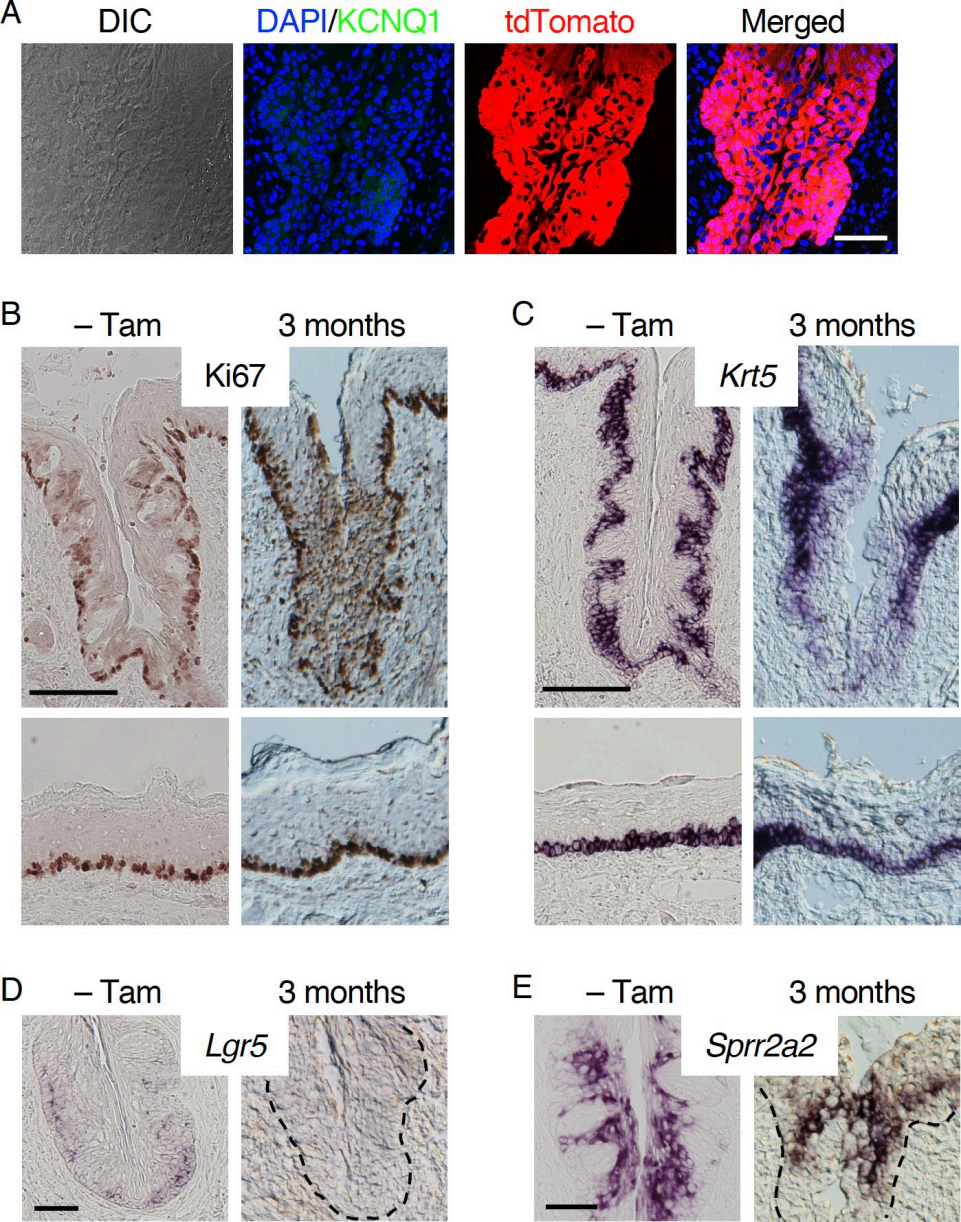
Long-term impact of *Sox2* deletion in the CvP. **A:** Lineage tracing of *Sox2*-deleted stem/progenitor cells together with immunohistochemical staining of KCNQ1 in the CvP of *Krt5*^*CreERT2/+*^; *Sox2*^*flox/flox*^; *Rosa26*^*lsl-Tom/+*^ mice (n = 2) 3 months after tamoxifen injection. **B:** Immunohistochemical staining of Ki67 in the CvP of *Krt5*^*CreERT2/+*^; *Sox2*^*flox/flox*^ (n = 1) and *Krt5*^*CreERT2/+*^; *Sox2*^*flox/flox*^; *Rosa26*^*lsl-Tom/+*^ mice (n = 2) 3 months after tamoxifen injection. **C-E:** Expression of *Krt*5 (C), *Lgr5* (D), and *Sprr2a2* (E) in CvP of *Krt5*^*CreERT2/+*^; *Sox2*^*flox/flox*^; *Rosa26*^*lsl-Tom/+*^ mice (n = 3) after 3 months of tamoxifen injection. Mice without tamoxifen injection (–Tam) were used as controls. Dotted lines show the boundary of epithelium and connective tissue. Scale bars, 100 μm (B, C) and 50 μm (A, D, E).

Impaired taste bud cell generation and altered gene expression in non-gustatory epithelial cells were observed shortly after *Sox2* deletion in stem/progenitor cells. Next, we examined the long term effects of *Sox2* deficiency in the stem/progenitor cell population. Three months after the induction of *Sox2* deficiency in stem/progenitor cells, SOX2 and KCNQ1 immunoreactive signals, as expected, were not detected in the CvP epithelium, while it was occupied by tdTomato^+^ cells (Figs 6A and S3). Similar to what we observed 7 days or later after the induction of *Sox2* deletion (Fig 1), the epithelial structure of CvP trench was disarranged: The boundary of epithelium and connective tissue was barely distinguishable in the bright field images, and significant immunoreactive signals to Ki67 were not confined to the base of epithelium but were distributed in the superficial (middle-to-apical) epithelial layers of the CvP, although Ki67 immunoreactive signals in non-papillary epithelia outside CvP and papillary apex of the CvP of *Sox2*-deleted mice were comparable to that in control mice (Fig 6B). Additionally, *Krt5* mRNA signals were also disorganized (Fig 6C). As predicted, *Sox2*-deleted stem/progenitor cells ceased to express *Lgr5* (Fig 6D). However, *Sprr2a2* expression was unexpectedly observed in the CvP epithelium (Fig 6E), indicating that the expression of *Sprr2a2* was only acutely and transiently affected by *Sox2* deletion. In addition, the *Sprr2a2* signal in the bottom half of trench was weaker than that in the top half and was observed sporadically in the CvP apex and/or just outside CvP. In comparison, the *Sprr2a2* signal showed a confined but throughout expression in the CvP trench in control mice. Long-term *Sox2* deficiency demonstrates the necessity of *Sox2* for generating taste bud cells and for organizing epithelial structure in the CvP. Recovered *Sprr2a2* expression suggests that *Sox2*-deficient stem/progenitor cells changed their nature and acquired compensatory mechanism for generating WT-like non-gustatory epithelial cells in the CvP during three month-long *Sox2* deficiency.

### *Sox2* is dispensable for homeostasis of non-gustatory papillae in the posterior tongue

**Fig 7:**
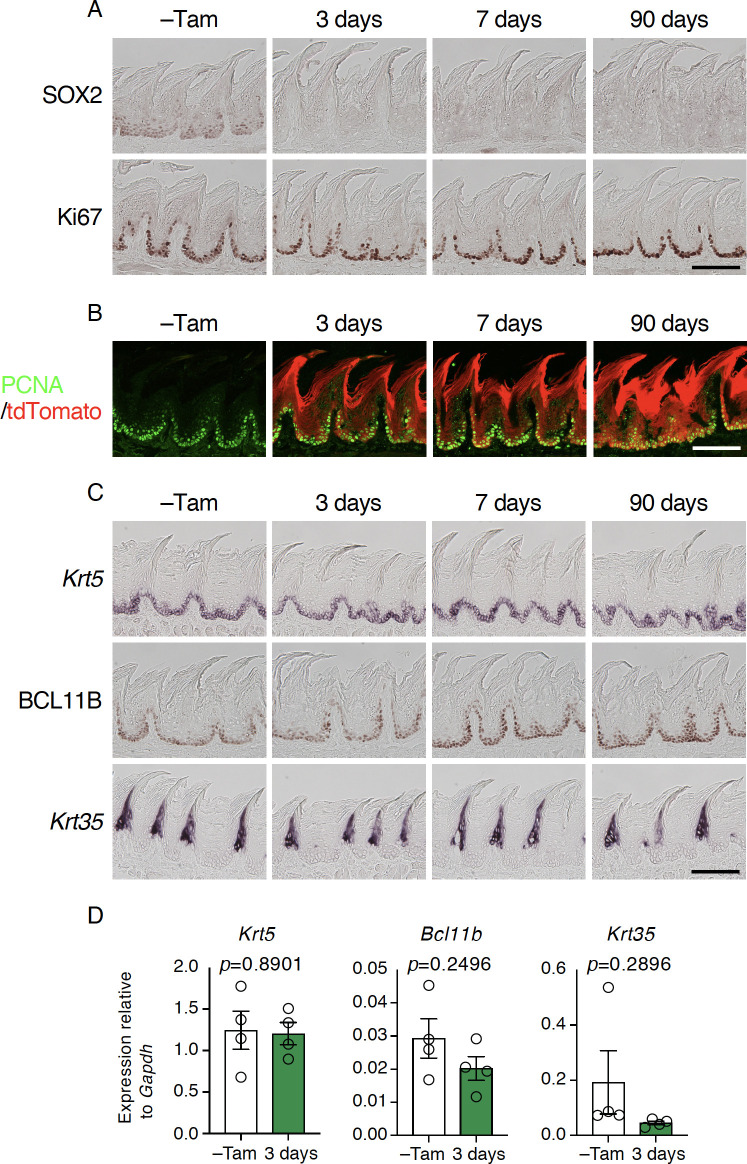
*Sox2* is dispensable for the normal turnover of non-gustatory papillary epithelial cells. **A:** Immunohistochemical staining of SOX2 (*top*) and Ki67 (*bottom*) in filiform papillae (FiP) in the intermolar eminence with and without tamoxifen injection (–Tam, control). **B:** Lineage tracing by tdTomato induced concurrently with *Sox2* deletion in stem/progenitor cells. Immunoreactive signal to PCNA (green) and tdTomato epifluorescence (red) are overlaid. **C:** Expression of marker genes and protein expressed in epithelial cells at distinct differentiation stages.Mice used are *Krt5*^*CreERT2/+*^; *Sox2*^*flox/flox*^ (n = 2 for–Tam, 3 days, and 7 days) and *Krt5*^*CreERT2/+*^; *Sox2*^*flox/flox*^; *Rosa26*^*lsl-Tom/+*^ mice (n = 1 for–Tam, 3 days, and 7 days; n = 3 for 90 days). Scale bars, 50 μm. **D:** Quantitative PCR analyses to evaluate epithelial cell marker gene expression in FiP in the intermolar eminence. Relative gene expression levels were normalized using *Gapdh* and statistically evaluated by Welch’s t-test (n = 4 each, *Krt5*^*CreERT2/+*^; *Sox2*^*flox/flox*^ mice before and 3 days after tamoxifen injection).

Lastly, we asked if *Sox2* is involved in the homeostatis of FiP, non-gustatory papillae that cover the most area of the dorsal surface of the tongue. Weak SOX2 immunoreactivity was observed at the base of epithelial layer where proliferating cells reside (Figs 7A and S2) in the control mice. After tamoxifen injection, SOX2 immunoreactivity was no longer detected in the FiP in *Krt5*^*CreERT2/+*^; *Sox2*^*flox/flox*^ and *Krt5*^*CreERT2/+*^; *Rosa26*^*lsl-Tom/+*^*; Sox2*^*flox/flox*^ mice, but no obvious change was found in the frequencies or distribution of immunoreactive singals to Ki67 or PCNA or mRNA expression of stem cell-specific keratin genes *Krt5* or *Krt14* in the FiP (Figs 7 and S4). Lineage tracing of *Sox2*-deleted stem/progenitor cells revealed continuous generation of epithelial cells, and the morphology of the FiP appeared normal after the *Sox2* deletion, even after three months (Fig 7A). In agreement, *Krt35* and *Krt84* (hard keratin genes expressed in the differentiated keratinocytes) and *Pax9* and BCL11B (transcription factors required for keratinocyte differentiation) [35, 36] appeared normal in intensity and distribution (Figs 7C and S4A). Quantitative RT-PCR also showed no significant difference of *Pax9*, *Bcl11b*, or *Krt35* expression by *Sox2* deletion, although *Krt84* mRNA expression was significantly decreased (Figs 7D and S4B). The machinery to regulate *Krt84* expression and/or its stability in the filiform keratinocytes may be affected by *Sox2* deficiency in their stem/progenitor cells. These results indicate that *Sox2* deficiency is dispensable for the homeostasis of posterior tongue epithelium where it is covered by FiP.

## Discussion

Single epithelial stem/progenitor cells in the gustatory papillae are tricompetent to generate cells; gustatory cells housed in taste buds, non-gustatory epithelial cells filling outside taste buds in the CvP epithelium, and themselves [37]. Likewise, epithelial stem/progenitor cells in the non-gustatory FiP are bicompetent to become papillary keratinocytes and copy themselves. SOX2 is widely expressed in oral epithelial stem/progenitor cells, and in this paper we demonstrate its role in the homeostatic regulation of posterior tongue epithelium. SOX2 is required for stem/progenitor cells to differentiate into gustatory-lineage cells but is dispensable for the viability and self-renewal of stem/progenitor cells and the homeostasis of epithelial cells in the non-gustatory FiP. SOX2 is also necessary for the normal turnover of non-gustatory CvP epithelium.

### Regional difference of requirement of SOX2 in stem/progenitor cells

SOX2 is expressed in stem/progenitor cells of many adult tissues [16, 18–20], albeit with distinct functions. For instance, SOX2 is required for the neurogenesis and self-renew of neural stem/progenitor cells in hippocampus [16], while it is dispensable for the self-renewal of stem/progenitor cells and the epithelial homeostasis in stomach and other tissues [38]. Even in the tongue epithelium, the function of SOX2 seems to be regionally distinct. SOX2 is required for the homeostasis of FiP in the anterior tongue [23] but not in the posterior tongue (this study). *Sox2* deletion stopped differentiation and increased the stem/progenitor cell population in FuP in the anterior tongue [23], whereas no significant increase in the number of stem/progenitor cells was observed in the CvP in the posterior tongue (this study). FuP and CvP are derived from the ectoderm and endoderm, respectively [9, 39], and *Sox2*^+^ adult stem/progenitor cells are derived from embryonic *Sox2*^+^ cells [18]. The regional differences of in the role of SOX2 in the tongue epithelial stem/progenitor cells may be ascribed to their distinct embryonic origins. However, intermolar eminence, where we examined the impact of *Sox2* deletion on FiP formation and noted no or little effects of SOX2 on FiP formation, is also derived from the ectoderm [9]. This suggests that aside from the embryonic origin, other factors may contribute to the regionally distinct roles of SOX2 in the function of lingual epithelial stem/progenitor cells, which would possibly be related to the differential expression of *Lgr5* between anterior and posterior gustatory papillae (i.e. FuP and CvP). In addition, *Lgr5* is not expressed in the stem/progenitor cells outside CvP even in the posterior tongue [10, 11]. Epithelial stem/progenitor cells in the tongue would be rather heterogeneous than homogeneous.

### Roles of SOX2 in epithelial stem/progenitor cells

*Sox2* deficiency did not perturb the generation of non-gustatory epithelial cells or renewal of stem/progenitor cells in FiP in posterior tongue. Also, no overt abnormalities were found in FiP by *Sox2* deficiency with regard to the morphology or molecular feature, reminiscent of the recent finding that *Sox2* is dispensable for the homeostasis of stomach and other epithelial tissues [38]. There may be a compensatory mechanism for *Sox2* deficiency in those epithelial stem/progenitor cells. Unlike FiP or other epithelial tissues, *Sox2* is necessary for the normal turnover of the CvP epithelium. Non-gustatory epithelial cells in the CvP trench were generated from *Sox2*-deleted stem/progenitor cells, but *Sprr2a2* expression was diminished throughout the CvP trench shortly after *Sox2* deficiency and recovered but unevenly distributed in the CvP trench afterwards. During chronic deficiency of SOX2, the function of SOX2 may be compensated by other unknown proteins, probably as in other epithelial stem/progenitor cells.

The most remarkable effect of *Sox2* deficiency in the posterior tongue epithelium is the loss of taste buds. Lineage tracing of *Sox2*-deleted stem/progenitor cells showed that *Sox2* deletion led to a remarkable decrease in the number of newly generated basal precursor cells in taste buds. Probably, stem/progenitor cells involve SOX2 in their fate determination toward gustatory lineage (i.e. taste bud) cells. At present, it is unclear what happens to *Sox2*-deleted stem/progenitor cells that would have become taste bud cells. They may change their fate to non-gustatory epithelial cells. There are other possibilities that impaired differentiation toward taste bud cells could increase stem/progenitor cell population as observed in the FuP [23], and *Sox2* deletion could have slowed cell cycle as inhibition of SOX2 reduced proliferation of neural stem/progenitor cells [40]. These possibilities are compatible with each other and might have occurred in the CvP so that *Sox2*-deleted stem/progenitor cell population were unaltered. It is interesting to study the differences in gene expression by RNA-seq and/or ChIP-seq between wild type and *Sox2*-deficient stem/progenitor cells.

## Methods

### Animals

C57B6/J (stock no. 000664), B6N.129S6(Cg)-*Krt5*^*tm1*.*1(cre/ERT2)Blh*^/J (*Krt5*^*CreERT2/+*^, stock no. 029155) [41], *Sox2*^*tm1*.*1Lan*^/J (*Sox2*^*flox/flox*^, stock no. 013093) [42], and B6.Cg-*Gt(ROSA)26Sor*^*tm14(CAG-tdTomato)Hze*^/J *(Rosa26*^*lsl-Tom/lsl-Tom*^, stock no. 007908) [43] mice were purchased from the Jackson Laboratory. Six weeks to 6 months old mice from both sexes were used for analyses. All animal experiments were approved by the Institutional Animal Care and Use Committee of the Monell Chemical Senses Center and performed in accordance with National Institutes of Health guidelines.

### Tamoxifen administration

Tamoxifen (10 mg/ml in corn oil; Sigma-Aldrich) was intraperitoneally injected to mice (100 mg/kg body weight), once for short-term chase (1–15 days) or five times over 5 consecutive days for long-term chase (≥2 months).

### Tissue preparation

Mice were deeply anesthetized with urethane and transcardially perfused with PBS followed by 4% paraformaldehyde (PFA) in PBS. Oral epithelia were dissected, postfixed, cryoprotected, and frozen as described previously [25]. To prepare fresh-frozen tissue samples, mice were deeply anesthetized with urethane, and oral epithelia were dissected and embedded in the frozen O.C.T. compound (Sakura Finetech). Cryosections (8 μm thickness) were prepared using a Leica CM1900 cryostat (Leica Microsystems), mounted on tissue-adhesive-coated glass slides (Fisher Scientific), and preserved in at -80˚C until use.

### Immunohistochemistry

Immunohistochemistry using 4% PFA-fixed sections was carried out as described previously [27]. The sections were treated in a preheated target retrieval solution (pH 9) (Agilent Technologies) at 80°C for 20 min before blocking. Primary antibodies were rabbit anti-KCNQ1 (Millipore, AB5932, 1:1000), goat anti-KCNQ1 (Santa Cruz Biotechnology, sc-10646, 1:300), goat anti-SOX2 (Santa Cruz Biotechnology, sc-17320, 1:300), rabbit anti-POU2F3 (Santa Cruz Biotechnology, sc-330, 1:1000), rabbit anti-DDC (GeneTex, GTX30448, 1:2000), mouse anti-PCNA (Millipore, NA03, 1:100), rabbit anti-active CASP3 (BD Biosciences, 559565, 1:500), rabbit anti-Ki67 (Abcam, ab15580, 1:1500), and rat anti-BCL11B (Abcam, ab18465, 1:1,000) antibodies. For single staining, a biotin-conjugated anti-rabbit IgG antibody, anti-goat IgG antibody, or anti-rat IgG antibody (Vector Laboratories, BA-1000, BA-9500, or BA-9400, 1:500) followed by an avidin-biotin complex (Vector Laboratories) was used as a secondary antibody, and signals were developed using the Metal Enhanced DAB Substrate Kit (Thermo Fisher Scientific). Details about antibodies used in this study are provided in S1 Table. For fluorescent labeling, Alexa 488-, 555-, and 647-conjugated antibodies (Thermo Fisher Scientific, A11029, A11034, A11055, A21206, A21432, A21447, A31572, 1:500) were used as secondary antibodies. Single-plane confocal images were acquired by a Leica TCS SP2 confocal microscope (Leica Microsystems) with a pinhole size of 1.5 airy units. Optical confocal images were merged, and brightness and contrast were optimized with Photoshop (Adobe Systems) and analyzed on a computer screen. Three to four sections were used per mouse in every experiment.

For quantitative analyses, signals per trench of CvP were counted from every 12 sections. SOX2, active CASP3, and tdTomato signals in taste buds were identified as those within KCNQ1^+^ cell clusters. SOX2, POU2F3, and DDC signals were normalized by their means in mice without tamoxifen injection.

### *In situ* hybridization

Single-label *in situ* hybridization using fresh-frozen sections of CvP or 4% PFA-fixed sections of FiP was carried out as described previously [27]. Digoxigenin-labeled antisense RNAs were used as probes after fragmentation to about 150 bases under alkaline conditions (see S2 Table for probe regions). Stained images were obtained using a Nikon eclipse 80i microscope equipped with a DXM1200C digital camera (Nikon Instruments) or BX-51 microscope equipped with a DP-71 digital camera (Olympus). For the combination of *in situ* hybridization and immunohistochemistry, 4% PFA-fixed sections were used. Sections were treated in a preheated target retrieval solution pH 9 (Dako) at 80˚C for 20 min, postfixed with 4% PFA in PBS, acetylated with acetic anhydride, prehybridized with salmon testis DNA, and hybridized with riboprobes. After washing, immunohistochemistry was performed, and then the fluorescent signals of riboprobes were developed using an alkaline phosphatase-conjugated anti-digoxigenin antibody (Roche Diagnostics, 11093274910, 1:500) and the HNPP Fluorescent Detection Set (Roche Diagnostics). Fluorescent images were acquired as described above. Three to four sections were used per mouse in every experiment (n≥3).

### Quantitative PCR

Epithelia of CvP and FiP in the posterior tongue were dissected from tongues of *Krt5*^*CreERT2/+*^; *Sox2*^*flox/flox*^ mice before and 3 days after tamoxifen injection as described previously [25]. Total RNA was extracted using TRIzol (Life Technologies, 15596026), and cDNA was synthesized using SuperScript™ IV VILO™ Master Mix with ezDNase™ Enzyme (Life Technologies, 11766050). Quantitative PCR was performed using Fast SYBR Green Master Mix Kit (Applied Biosystems, 4385612). *Gapdh* was used as control to normalize the expression levels of individual genes. The relative gene expression level was calculated as 2^-(CTTarget-CTGapdh)^ [44]. Details about the primers used are shown in S3 Table.

### Statistical analyses

Data are shown as the mean ± s.e.m. Following statistical analyses were conducted using Prism 6 (GraphPad Software). A Welch’s ANOVA followed by Dunnett’s T3 multiple comparisons test was used to determine the effects over time by *Sox2* deletion on marker protein expression. Welch’s *t*-test was used to determine the effect of *Sox2* deletion on the mRNA expression in the CvP and FiP.

## Supporting information

S1 Fig *Krt5* mRNA is predominantly expressed in epithelial stem/progenitor cells in the oral cavity and not in taste buds. **A:** Distribution of *Krt5* mRNA expression signal in the tongue epithelium of wild type mice. *Krt5* was expressed in the basal epithelial cells in the gustatory (circumvallate papillae, CvP; fungiform papillae, FuP; and soft palate) and non-gustatory epithelia surrounding CvP (n = 1, 3 sections for CvP; 9 sections for FuP and soft palate). **B:** Triple fluorescent labeling of the pan-taste-bud-cell marker KCNQ1 (blue), the proliferation cell marker PCNA (green), and *Krt5* mRNA (red) in the CvP (*left*) and surrounding non-gustatory epithelia (*right*) of wild type mice (n = 3). **C:** Triple fluorescent labeling of SOX2 (green), PCNA (blue), and *Krt5* mRNA (red) in the CvP of wild type mice (n = 3). **D, E:** Lineage tracing of *Krt5*-expressing cells in the CvP (D) and non-papillary epithelia surrounding CvP (E) of *Krt5*^*CreERT2/+*^; *Rosa26*^*lsl-Tom/+*^ mice with and without tamoxifen injection (–Tam, control). Fluorescences indicates KCNQ1 immunoreactive signals (green) and tdTomato (red). N = 2,–Tam; n = 1, 1 day; n = 4, 3 days; n = 3, 2 and 6 months. **F:** Examples of sporadic spontaneous tdTomato expression in tongue epithelium of tamoxifen-untreated *Krt5*^*CreERT2/+*^; *Rosa26*^*lsl-Tom/+*^ mice. Scale bars, 50 μm.
(TIF)

S2 Fig *Sox2* deletion and SOX2 immunoreactivity in the non-papillary epithelium surrounding CvP. Immunoreactive signals to SOX2 were present in the nuclei of the basal cells in the epithelium of *Krt5*^*CreERT2/+*^; *Sox2*^*flox/flox*^ mice without tamoxifen injection. After tamoxifen injection, such signals were not detected. N = 2, 3 months; n = 3,–Tam, 3 days, and 1 week. Scale bar, 50 μm.
(TIF)

S3 Fig Loss of immunoreactivities to SOX2 and KCNQ1 long after the *Sox2* deletion in stem/progenitor cells. Immunohistochemical staining of SOX2 (*top*) and KCNQ1 (*bottom*) in circumvallate papillae (CvP) of *Krt5*^*CreERT2/+*^; *Sox2*^*flox/flox*^ mice 3 months after tamoxifen injection (*right*) and without tamoxifen injection (*left*). The broken lines show the boundary of epithelium and connective tissue. N = 3. Scale bar, 50 μm.
(TIF)

S4 Fig Gene expression in non-gustatory papillary epithelial cells. **A:** *In situ* hybridization analyses of a stem cell-specific *Krt14* (*top*), a transcription factor *Pax9* (*middle*) required for FiP morphogenesis, and a hard keratin *Krt84* (*bottom*) in sagittal sections of the intermolar eminence in posterior dorsal tongue. Mice used are *Krt5*^*CreERT2/+*^; *Sox2*^*flox/flox*^ (n = 2 for–Tam, 3 days, and 7 days) and *Krt5*^*CreERT2/+*^; *Sox2*^*flox/flox*^; *Rosa26*^*lsl-Tom/+*^ mice (n = 1 for–Tam, 3 days, and 7 days; n = 3 for 90 days). Scale bars, 50 μm. **B:** Quantitative PCR analyses to evaluate the expression of epithelial cell marker genes in FiP in the intermolar eminence in *Krt5*^*CreERT2/+*^; *Sox2*^*flox/flox*^ mice 3 days after tamoxifen injection and without tamoxifen injection (–Tam, control) (n = 4 each). Relative gene expression levels were normalized using *Gapdh* and statistically evaluated by Welch’s t-test.
(TIF)

S1 Table Antibodies used for immunohistochemistry and *in situ* hybridization analyses. (PDF)

S2 Table Probes used for *in situ* hybridization analyses. (PDF)

S3 Table Information on primers used for qPCR analyses. (PDF)
