# Supplementary figures and images for: SOX2 regulates homeostasis of taste bud cells and lingual epithelial cells in posterior tongue
Source: PLoS One. 2020 Oct 15;15(10):e0240848. doi: 10.1371/journal.pone.0240848 (PMC7561181; doi:10.1371/journal.pone.0240848)

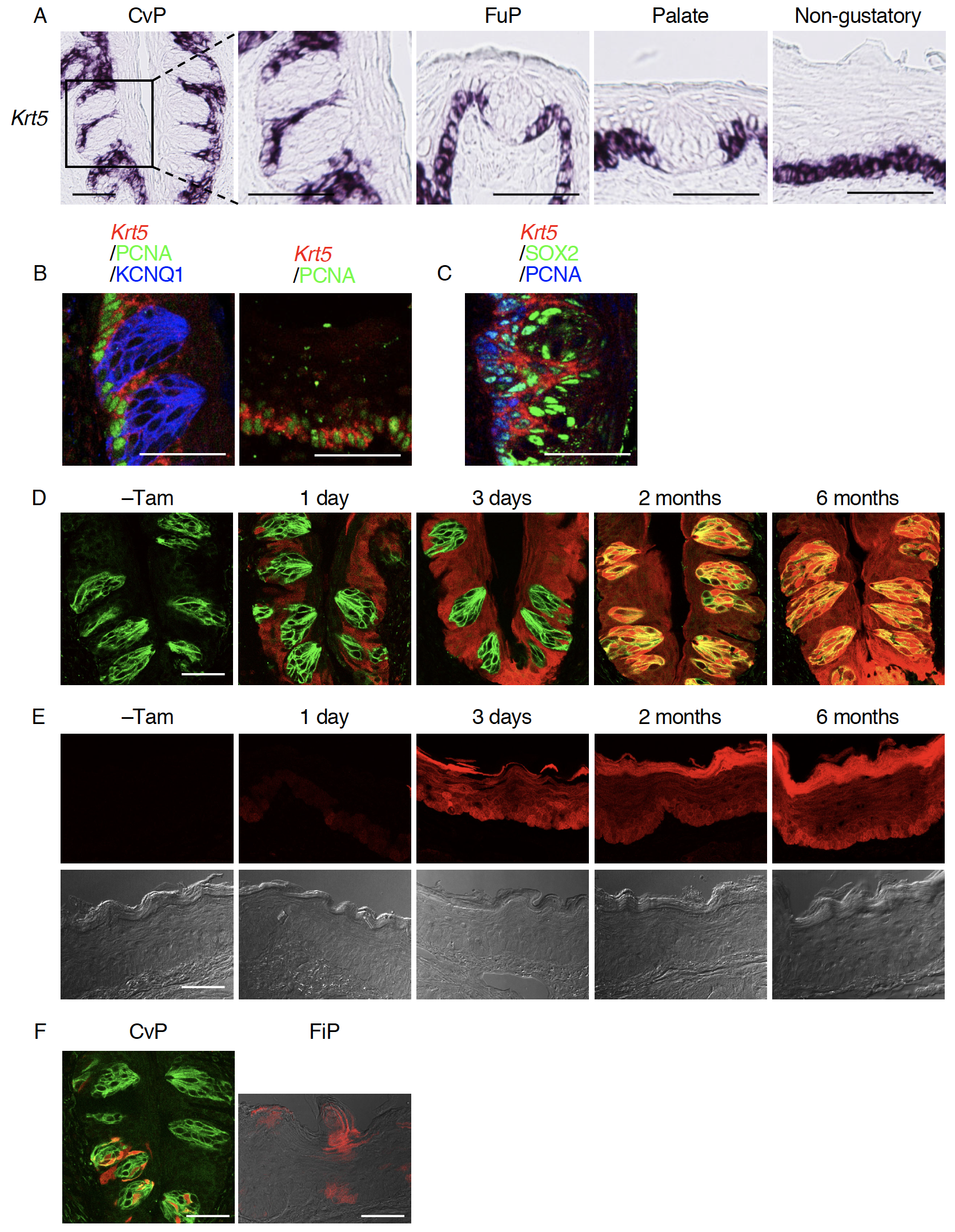

Supplement: S1 Fig — A: Distribution of Krt5 mRNA expression signal in the tongue epithelium of wild type mice. Krt5 was expressed in the basal epithelial cells in the gustatory (circumvallate papillae, CvP; fungiform papillae, FuP; and soft palate) and non-gustatory epithelia surrounding CvP (n = 1, 3 sections for CvP; 9 sections for FuP and soft palate). B: Triple fluorescent labeling of the pan-taste-bud-cell marker KCNQ1 (blue), the proliferation cell marker PCNA (green), and Krt5 mRNA (red) in the CvP (left) and surrounding non-gustatory epithelia (right) of wild type mice (n = 3). C: Triple fluorescent labeling of SOX2 (green), PCNA (blue), and Krt5 mRNA (red) in the CvP of wild type mice (n = 3). D, E: Lineage tracing of Krt5-expressing cells in the CvP (D) and non-papillary epithelia surrounding CvP (E) of Krt5CreERT2/+; Rosa26lsl-Tom/+ mice with and without tamoxifen injection (–Tam, control). Fluorescences indicates KCNQ1 immunoreactive signals (green) and tdTomato (red). N = 2,–Tam; n = 1, 1 day; n = 4, 3 days; n = 3, 2 and 6 months. F: Examples of sporadic spontaneous tdTomato expression in tongue epithelium of tamoxifen-untreated Krt5CreERT2/+; Rosa26lsl-Tom/+ mice. Scale bars, 50 μm. (TIF) [file pone.0240848.s001.tif]

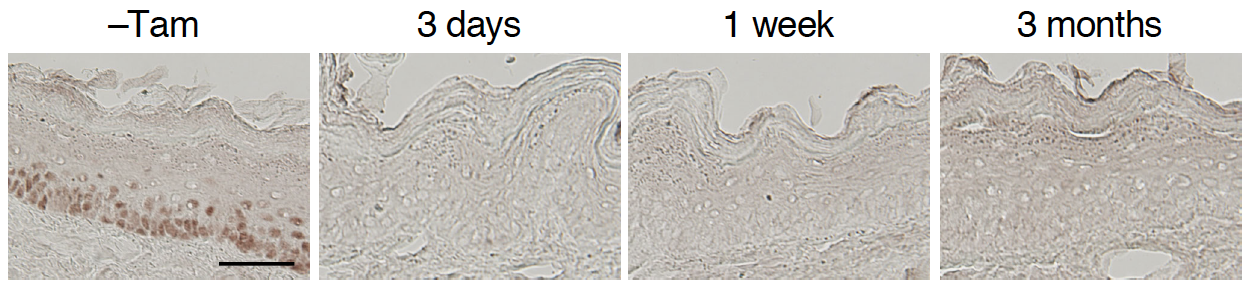

Supplement: S2 Fig — Immunoreactive signals to SOX2 were present in the nuclei of the basal cells in the epithelium of Krt5CreERT2/+; Sox2flox/flox mice without tamoxifen injection. After tamoxifen injection, such signals were not detected. N = 2, 3 months; n = 3,–Tam, 3 days, and 1 week. Scale bar, 50 μm. (TIF) [file pone.0240848.s002.tif]

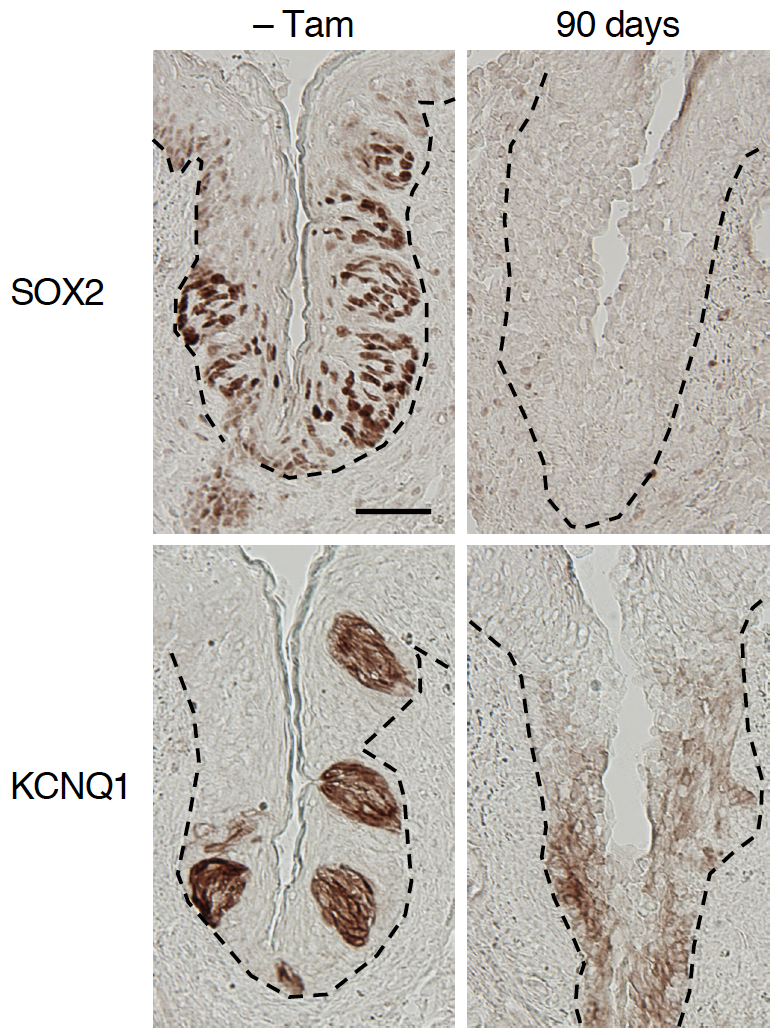

Supplement: S3 Fig — Immunohistochemical staining of SOX2 (top) and KCNQ1 (bottom) in circumvallate papillae (CvP) of Krt5CreERT2/+; Sox2flox/flox mice 3 months after tamoxifen injection (right) and without tamoxifen injection (left). The broken lines show the boundary of epithelium and connective tissue. N = 3. Scale bar, 50 μm. (TIF) [file pone.0240848.s003.tif]

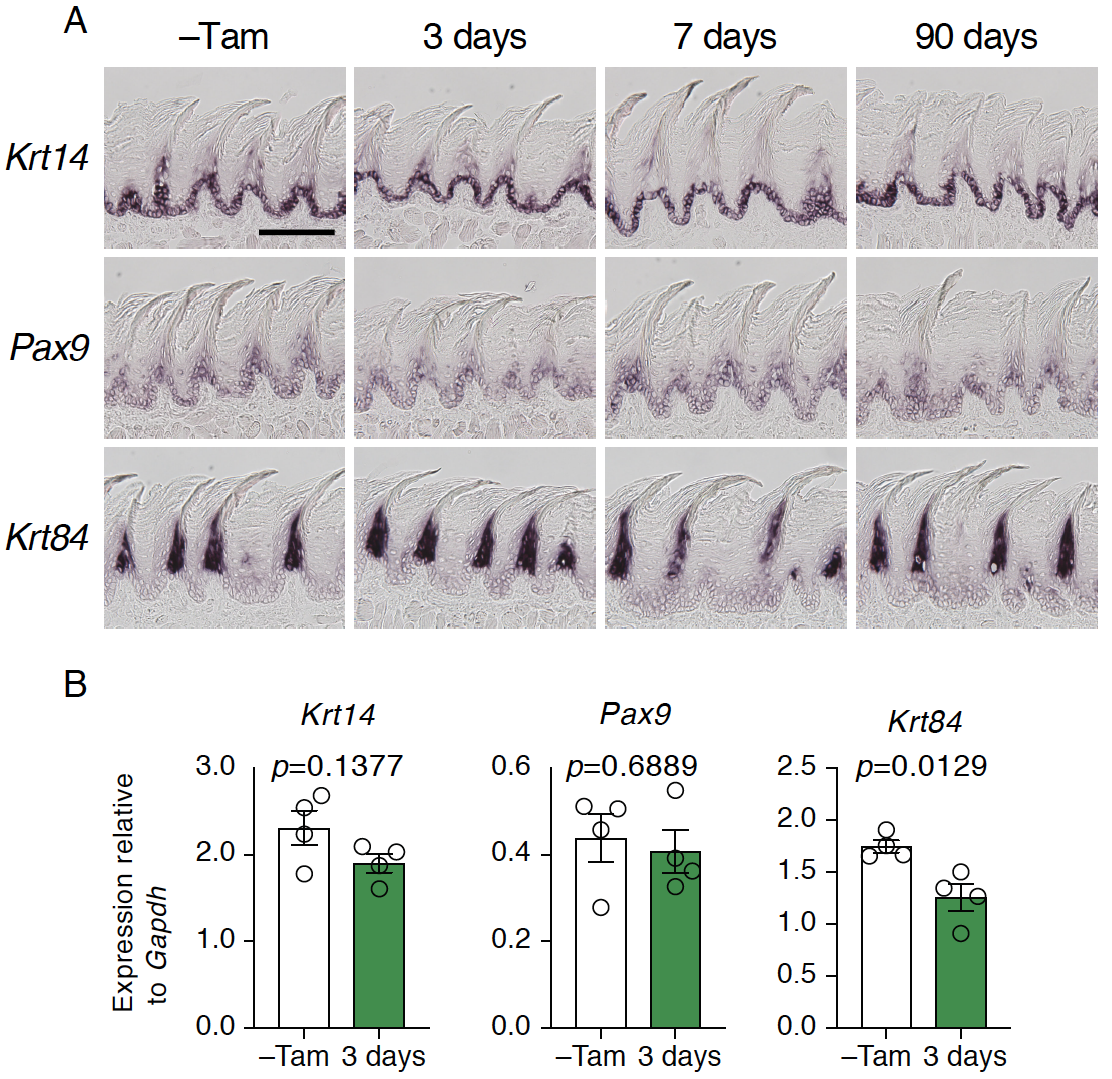

Supplement: S4 Fig — A: In situ hybridization analyses of a stem cell-specific Krt14 (top), a transcription factor Pax9 (middle) required for FiP morphogenesis, and a hard keratin Krt84 (bottom) in sagittal sections of the intermolar eminence in posterior dorsal tongue. Mice used are Krt5CreERT2/+; Sox2flox/flox (n = 2 for–Tam, 3 days, and 7 days) and Krt5CreERT2/+; Sox2flox/flox; Rosa26lsl-Tom/+ mice (n = 1 for–Tam, 3 days, and 7 days; n = 3 for 90 days). Scale bars, 50 μm. B: Quantitative PCR analyses to evaluate the expression of epithelial cell marker genes in FiP in the intermolar eminence in Krt5CreERT2/+; Sox2flox/flox mice 3 days after tamoxifen injection and without tamoxifen injection (–Tam, control) (n = 4 each). Relative gene expression levels were normalized using Gapdh and statistically evaluated by Welch’s t-test. (TIF) [file pone.0240848.s004.tif]
