# Supplementary material for: SOX2 regulates homeostasis of taste bud cells and lingual epithelial cells in posterior tongue
Source: PLoS One. 2020 Oct 15;15(10):e0240848. doi: 10.1371/journal.pone.0240848 (PMC7561181; doi:10.1371/journal.pone.0240848)
Supplement: S1 Table — (PDF) [file pone.0240848.s005.pdf]

Table S1. Antibodies used for immunohistochemistry and *in situ* hybridization analyses.

Antibody	Manufacture	Catalog No.	RRID number
Rabbit anti-KCNQ1	Millipore	AB5932	RRID:AB_92147
Goat anti-KCNQ1	Santa Cruz Biotechnology	sc-10646	RRID:AB_2131554
Goat anti-SOX2	Santa Cruz Biotechnology	sc-17320	RRID:AB_2286684
Rabbit anti-POU2F3	Santa Cruz Biotechnology	sc-330	RRID:AB_677443
Rabbit anti-DDC	GeneTex	GTX30448	RRID:AB_367199
Mouse anti-PCNA	Millipore	NA03	RRID:AB_2160355
Rabbit anti-active CASP3	BD Biosciences	559565	RRID:AB_397274
Rabbit anti-Ki67	Abcam	ab15580	RRID:AB_443209
Rat anti-BCL11B	Abcam	ab18465	RRID:AB_2064130
Biotin-conjugated Goat anti-rabbit IgG	Vector Laboratories	BA-1000	RRID:AB_2313606
Biotin-conjugated Horse anti-goat IgG	Vector Laboratories	BA-9500	RRID:AB_2336123
Biotin-conjugated Goat anti-rat IgG	Vector Laboratories	BA-9400	RRID:AB_2336202
Alexa488-conjugated Goat anti-mouse IgG	Thermo Fisher	A11029	RRID:AB_2534088
Alexa488-conjugated Goat anti-Rabbit IgG	Thermo Fisher	A11034	RRID:AB_2576217
Alexa488-conjugated Donkey anti-Goat IgG	Thermo Fisher	A11055	RRID:AB_2534102
Alexa488-conjugated Donkey anti-Rabbit IgG	Thermo Fisher	A21206	RRID:AB_2535792
Alexa555-conjugated Donkey anti-Goat IgG	Thermo Fisher	A21432	RRID:AB_2535853
Alexa647-conjugated Donkey anti-Goat IgG	Thermo Fisher	A21447	RRID:AB_2535864
Alexa555-conjugated Donkey anti-Rabbit IgG	Thermo Fisher	A31572	RRID:AB_162543
Alkaline phosphatase-conjugated Sheep anti-digoxigenin	Roche Diagnostics	11093274910	RRID:AB_514497
