# Supplementary material for: SOX2 regulates homeostasis of taste bud cells and lingual epithelial cells in posterior tongue
Source: PLoS One. 2020 Oct 15;15(10):e0240848. doi: 10.1371/journal.pone.0240848 (PMC7561181; doi:10.1371/journal.pone.0240848)
Supplement: S2 Table — (PDF) [file pone.0240848.s006.pdf]

Table S2. Probes used for *in situ* hybridization analyses.

Gene Name	Accession No.	Probe Region
Lgr5	BC156649	37-2760
Sprr2a2	BC010818	1-738
Entpd2	NM_009849	20-1822
Krt5	BC108361	1860-2253
Krt14	BC011074	1311-1680
Krt35	BC100542	1-1869
Krt84	BC114971	1-2161
Pax9	BC005794	1-1635
