# Supplementary material for: SOX2 regulates homeostasis of taste bud cells and lingual epithelial cells in posterior tongue
Source: PLoS One. 2020 Oct 15;15(10):e0240848. doi: 10.1371/journal.pone.0240848 (PMC7561181; doi:10.1371/journal.pone.0240848)
Supplement: S3 Table — (PDF) [file pone.0240848.s007.pdf]

Table S3. Information on primers used for qPCR analyses.

Gene	Accession No.	Amplified region	Forward primer	Reverse primer
Gapdh	BC085315	735-843	GCATGGCCTTCCGTGTTCCTA	GATGCCTGCTTCACCACCTTCT
Krt5	BC108361	1185-1288	TGGCGATGACCTTCGAAACA	GGTTGGCACACTGCTTCTTG
Krt14	BC011074	987-1140	GGTTCTTCAGCAAGACAGAGG	TCCAGGGATGCTTTCATGCTG
Lgr5	BC156649	1522-1720	TAAAGACGACGGCAACAGTG	GATTCGGATCAGCCAGCTAC
Sprr2a	BC010818	110-230	GAAGTGCCCTGAGCCTTGTCC	CTCATAGCACACTACAGGACG
Bcl11b	NM_001079883	2715-2839	AGTGCGAGCTGTGCAACTACG	GTAGACGCTGAAGGGCATCTG
Pax9	BC005794	880-1040	CACGCAGTGAATGGATTGGAG	GCAGCACTGTAGGTCATGTAG
Krt35	BC100542	896-1053	CTGGTATGACACCCAGACCGA	AGTCCAAAGCATCTCTCATGC
Krt84	BC114971	1396-1556	ATGAATGTCAAGCTGGCCCTG	CTGGATCCAGAGACTAACTCG
